# Low-Density Lipoprotein Cholesterol Gymnastics: Exploring the Advantages and Limitations of the Friedewald, Martin–Hopkins, and Sampson Equations for Personalized Lipid Management

**DOI:** 10.3390/jpm14091000

**Published:** 2024-09-20

**Authors:** Ion Bogdan Mănescu, Liliana Demian, Minodora Dobreanu

**Affiliations:** 1 Department of Laboratory Medicine, Faculty of Medicine, George Emil Palade University of Medicine, Pharmacy, Science, and Technology of Targu Mures, 38 Gheorghe Marinescu, 540142 Targu Mures, Romania; minodora.dobreanu@umfst.ro; 2 Doctoral School, George Emil Palade University of Medicine, Pharmacy, Science, and Technology of Targu Mures, 38 Gheorghe Marinescu, 540142 Targu Mures, Romania; 3 Clinical Laboratory, Emergency County Clinical Hospital of Targu Mures, 50 Gheorghe Marinescu, 540136 Targu Mures, Romania; lilidemian@yahoo.com; 4 Immunology Laboratory, Center for Advanced Medical and Pharmaceutical Research (CCAMF), George Emil Palade University of Medicine, Pharmacy, Science, and Technology of Targu Mures, 38 Gheorghe Marinescu, 540142 Targu Mures, Romania

**Keywords:** algorithm, calculation, estimation, Friedewald, low-density lipoprotein cholesterol, Martin–Hopkins, pattern, Sampson

## Abstract

Background: The most commonly used method for low-density lipoprotein cholesterol (LDL-C) estimation is the Friedewald equation, which has notable limitations. However, more accurate methods have been proposed. This study investigates the advantages and limitations of these methods and identifies the contexts in which each equation is the most or least applicable. Methods: A cohort of 222 individuals underwent a standard lipid profile assessment, including directly measuring their LDL-C (dLDL-C). LDL-C was also estimated using the Friedewald, Martin–Hopkins, and Sampson equations. The differences (%Delta) between the estimated and measured LDL-C were analyzed in relation to dLDL-C, high-density lipoprotein cholesterol (HDL-C), and triglyceride levels. Results: The %Delta was significantly lower (*p* < 0.0001) for the Martin–Hopkins (−8.8 ± 9.8) and Sampson (−9.5 ± 9.2) equations compared to Friedewald (−12.2 ± 9.2). All equations increasingly underestimated LDL-C as the dLDL-C levels decreased. The %Delta of the Martin–Hopkins equation showed significant positive correlations with dLDL-C (≤130 mg/dL) and triglycerides and a significant negative correlation with HDL-C. In a subgroup of 30 individuals with extreme %Delta values, patterns of gross underestimation were observed, particularly when low LDL-C, low triglycerides, and high HDL-C coincided. Conclusions: The Martin–Hopkins equation is a superior method for LDL-C estimation and a valuable tool in precision medicine. However, clinicians and laboratory professionals must be aware of its limitations and recognize patterns that could lead to significant LDL-C underestimation. We propose an algorithm for clinical laboratories to provide personalized LDL-C assessments.

## 1. Introduction

Although the limitations of low-density lipoprotein cholesterol (LDL-C) estimation equations have been well documented for many years, the most common method for determining LDL-C concentration remains calculation. The most popular method used for estimating LDL-C, almost monopolizing the field, is the well-known Friedewald equation [1]. However, the Friedewald equation has been shown to consistently underestimate LDL-C, sometimes significantly, with important clinical consequences [1,2,3,4,5,6,7,8]. This underestimation impacts the risk classification of patients, potentially resulting in calculated LDL-C below the therapeutic target, while the actual LDL-C remains above the target [3,4,5,6,7,8].

Given the inaccuracies of the Friedewald equation, especially at low LDL-C levels [1,2,3,4,5,6,7,8], researchers have developed more accurate equations for LDL-C estimation. The Sampson equation is a more recent and mathematically complex alternative to Friedewald [5]. Despite its increased accuracy [3,4,5], the Sampson equation still employs a “one size fits all” approach, as it is based on a single, unadjustable mathematical equation. In contrast, the more popular Martin–Hopkins equation [8], published over a decade ago, replaces the constant in the Friedewald equation (which is 5) with a variable factor determined from a table of 180 cells, each with its own value for the factor (ranging between 3.1 and 11.9). Because the factor is adjusted based on the patient’s triglyceride (TG) and non-high-density lipoprotein cholesterol (non-HDL-C) levels, the Martin–Hopkins equation can be considered a tool of personalized medicine.

While it is debated whether the Martin–Hopkins equation is superior to the Sampson equation, it is well established that both are significantly more accurate than the Friedewald equation. Despite consistent evidence of their superiority, neither the Martin–Hopkins nor the Sampson equations have been widely implemented in clinical laboratories.

In this study, we aimed to test and validate the Martin–Hopkins and Sampson equations in a mixed cohort. We also sought to explore their advantages and limitations to determine the specific contexts in which these equations should be used and the scenarios where they may be less applicable.

## 2. Materials and Methods

### 2.1. Study Design and Selection of Participants

This work is part of a larger study approved by the Ethics Committee of the Emergency Institute for Cardiovascular Diseases (approval no. 486/19.01.2017), and all procedures were performed according to the Declaration of Helsinki. All participants gave written informed consent for study participation. The study included a total of 222 participants, of which 125 had premature coronary heart disease and 97 were healthy individuals. The exclusion criteria for both patients and healthy individuals were as follows: (1) below 18 years of age and (2) any disease known to affect lipid metabolism, such as diabetes mellitus, chronic kidney disease, chronic liver disease, hypo- or hyperthyroidism, etc.

### 2.2. Lipid Profile Analysis

After an overnight fast, peripheral blood was collected in EDTA and serum separator tubes. The serum concentrations of total cholesterol (TC), high-density lipoprotein cholesterol (HDL-C), low-density lipoprotein cholesterol (LDL-C), and triglycerides (TGs) were measured on a Cobas Integra 400 plus analyzer (Hoffmann-La Roche, Basel, Switzerland) according to the manufacturer’s instructions, using commercially available kits (Roche; REFs 03039773, 04399803, 07005717, and 20767107, respectively). Non-HDL-C was calculated as the difference between TC and HDL-C. Plasma from EDTA tubes was separated, and lipoprotein(a) was measured by nephelometry on a BN ProSpec analyzer (Siemens Healthineers, Munich, Germany) according to the manufacturer’s instructions, using commercially available kits (Behring Diagnostics, London, UK; REF OQHL11).

The direct measurement of LDL-C (Roche REF 07005717) is performed using a homogeneous enzymatic colorimetric assay, which employs cholesterol esterase and cholesterol oxidase in the presence of surfactants that selectively solubilize LDL particles. This method is standardized against beta-quantification, which is the reference method. The assay’s measurement range extends from 3.87 mg/dL (LoB, LoD, LoQ) to 549 mg/dL. It complies with the 1995 NCEP guidelines, achieving less than 4% imprecision, less than 4% bias, and under 12% total analytical error. The assay is unaffected by elevated bilirubin, hemoglobin, lipemia, or common medications, including high doses of vitamin C. However, non-fasting samples may yield slightly lower LDL-C concentrations, as can EDTA plasma compared to the serum. Additionally, N-acetylcysteine may result in falsely low LDL-C levels, and paraproteinemia may produce unreliable results.

### 2.3. LDL-C Estimation and Statistical Analysis

LDL-C was measured directly (dLDL-C) and also estimated based on the Friedewald, Martin–Hopkins, and Sampson equations. The Friedewald equation [1] is as follows: LDL-C = TC − HDL-C − TG/5. The Martin–Hopkins equation is as follows: LDL-C = TC − HDL-C − TG/F. Here, F is an adjustable factor that depends on the TG and non-HDL-C levels and was retrieved from the original publication describing the equation [8]. The Sampson equation [5] is as follows: LDL-C = TC/0.948 − HDL-C/0.971 − [TG/8.56 + (TG × non-HDL-C)/2140 − TG2/16100] − 9.44.

For this study, dLDL-C was used as the reference method. The estimated concentrations were compared with dLDL-C using a %Delta metric calculated as follows: %Delta (%) = 100 × (Estimated LDL-C − Measured LDL-C)/Measured LDL-C. Therefore, %Delta was employed as a measure of bias for each equation relative to the reference (dLDL-C).

Statistical analyses were conducted using MedCalc^®^ Statistical Software version 20.104 (MedCalc Software Ltd., Ostend, Belgium. Data normality was assessed using the Kolmogorov–Smirnov test. Paired data tests (either Student’s *t*-test or the Wilcoxon test) were utilized to compare dLDL-C and estimated LDL-C. Pearson correlation analysis was used for correlation assessments. Statistical significance was established at *p* ≤ 0.05.

When comparing the estimated LDL-C levels with dLDL-C, the following thresholds were arbitrarily set: a %Delta between −5% and 5% was considered a negligible (non-significant) difference, a %Delta between −5% and −20% or between 5% and 20% was considered a significant difference, and a %Delta below −20% or above 20% was considered a critical difference. These thresholds were also applied when comparing %Delta values from different equations.

## 3. Results

Out of the 222 participants initially included, 12 were excluded due to hypertriglyceridemia (TG > 400 mg/dL). Consequently, the statistical analysis was conducted on 210 participants, of which 167 were male (79.5%) and 90 were undergoing statin treatment (42.8%). The average age of the cohort was 48.5 years (SD: 6.6 years). The blood lipid concentrations of the entire cohort were as follows: mean TC was 178.9 mg/dL (SD 43.7 mg/dL), median HDL-C was 43.4 mg/dL (IQR 36.0–53.4 mg/dL), median TG was 126.8 mg/dL (IQR 94.4–167.3 mg/dL), and mean non-HDL-C was 133.1 mg/dL (SD 41.8 mg/dL). 

The concentration of dLDL-C and its estimation using the three equations are presented in Table 1, along with the %Delta values for each equation. While there was no significant difference between the estimations made by the Martin–Hopkins and Sampson equations (*p* = 0.09), both were significantly different from the Friedewald estimation (*p* < 0.0001). A similar analysis, presented in Table 2 (upper part), was performed after dividing the cohort into two categories: those with (n = 90) and without statin treatment (n = 120). Consistent with the overall cohort analysis, in the untreated group, there was no statistical difference between the Martin–Hopkins and Sampson estimations, but both were significantly different from the Friedewald estimation (*p* < 0.0001). In the statin-treated group, the Friedewald estimation was once again inferior (*p* < 0.0001). However, the Martin–Hopkins estimation was also significantly superior to the Sampson estimation (*p* < 0.0001). In all analyzed scenarios (Table 1 and Table 2), the t-test revealed significant differences between dLDL-C and the estimated LDL-C for all three equations (*p* < 0.0001 for all comparisons).

**Figure 1:**
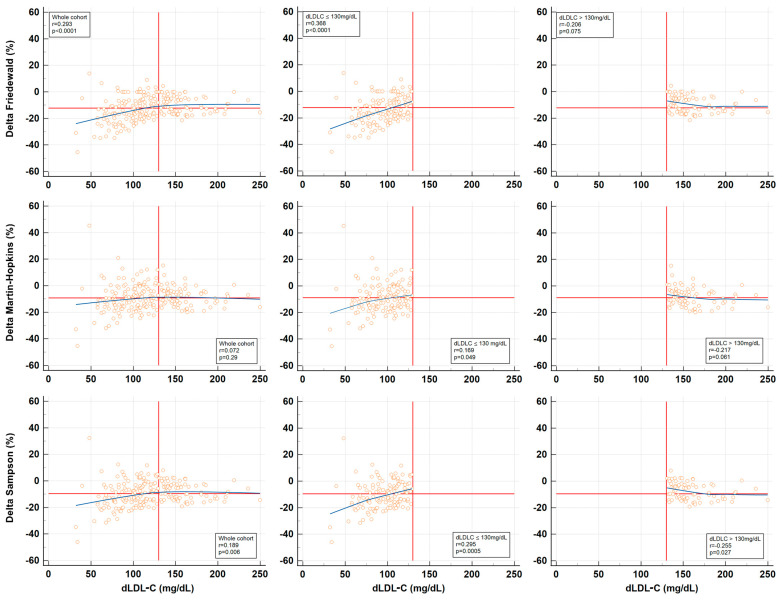
Correlations between %Delta values (the difference between estimated and measured LDL-C) and dLDL-C for each of the three equations studied. Initially, correlations were assessed for the entire cohort (n = 210). Due to the observed biphasic trend in the data, as indicated by the blue LOESS trend lines (smoothing span: 99%), the analysis was stratified by dLDL-C levels, dividing the dataset at the vertical red line into two subdomains: ≤130 mg/dL (n = 135) and >130 mg/dL (n = 75). The horizontal red line represents the mean %Delta value for each equation.

**Figure 2:**
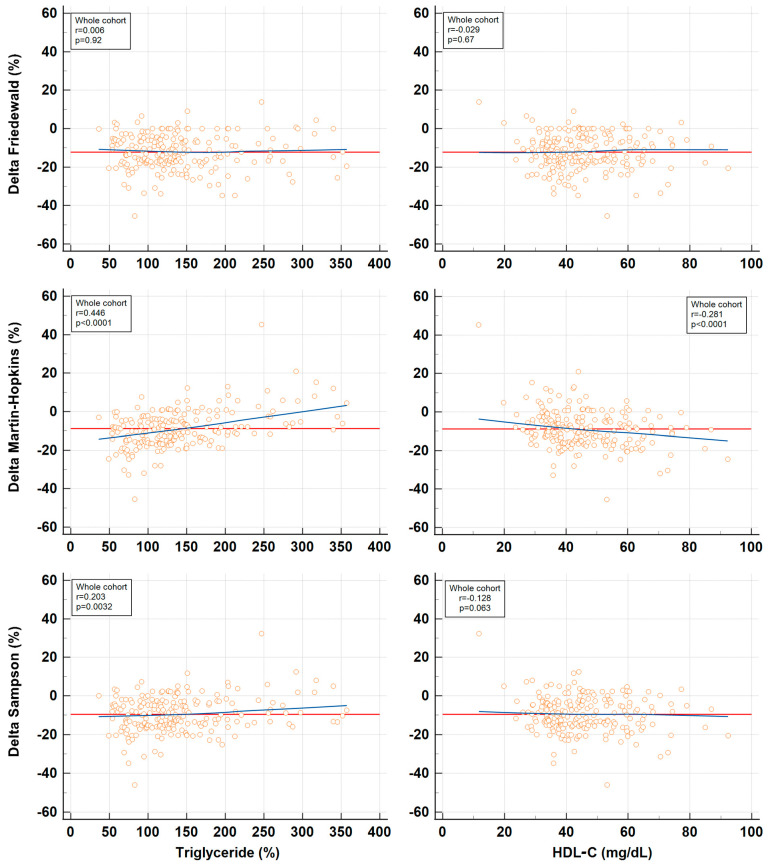
Correlations between %Delta values (the difference between estimated and directly measured LDL-C) and triglycerides or HDL-C for each of the three equations evaluated. The analysis is based on a cohort of 210 individuals. The data trends are illustrated by the blue LOESS trend lines (smoothing span: 99%). The horizontal red line indicates the mean %Delta value for each equation.

To examine how %Delta values, calculated for each equation, vary with the dLDL-C levels, correlation analyses were performed and are depicted in Figure 1. The %Delta values from all three equations exhibited a biphasic relationship with the dLDL-C levels. Consequently, the spectrum of dLDL-C values was divided into two subdomains based on visual indications, and the analysis was repeated for each subdomain: ≤130 mg/dL and >130 mg/dL (Figure 1). The numerical comparison of dLDL-C and LDL-C estimations by the three equations for these subdomains of dLDL-C is shown in the lower part of Table 2. Correlation analyses were also used to investigate how %Delta values, calculated for each equation, vary with the TG and HDL-C levels (Figure 2).

Based on the results of the statistical analysis, we concluded that each equation was prone to error, and the magnitude of that error was associated with blood levels of LDL-C, HDL-C, and TGs. Thus, we summarized our findings in Table 3, hypothesizing the perturbing factors that may skew the equations towards extreme misrepresentations of the dLDL-C concentration, which were either underestimation (most frequently) or overestimation (less commonly).

**Figure 3:**
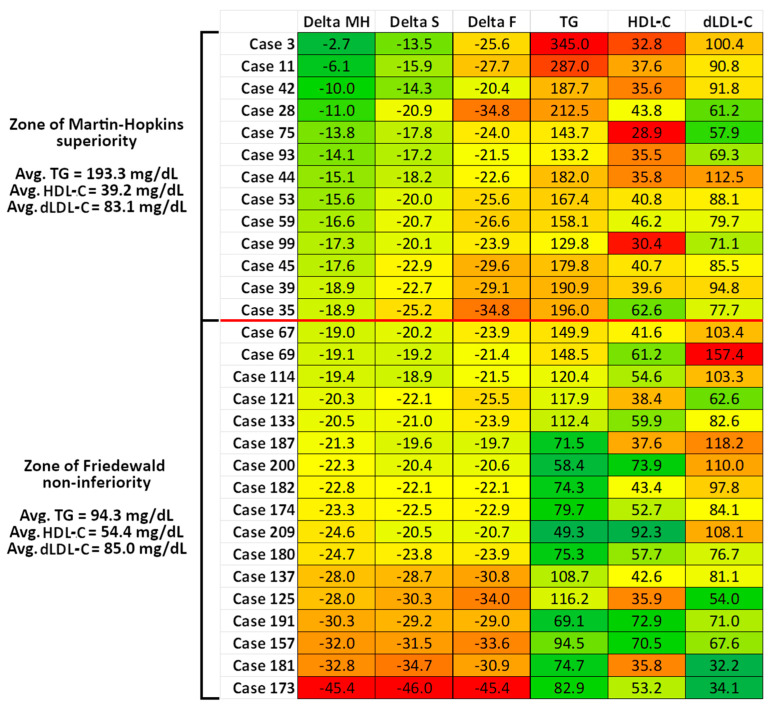
This heat map displays a cohort subgroup of 30 individuals, selected due to at least one extreme %Delta value (the difference between estimated and directly measured LDL-C: either below −20% or above 20%). The columns are indexed based on the %Delta Martin–Hopkins value, which is presented in the first column from least to most biased. %Delta values: green indicates low bias, while red signifies high bias. Triglycerides (TGs) and directly measured LDL-C (dLDL-C): green represents low concentrations and red indicates high concentrations. HDL-C: green denotes high concentrations and red denotes low concentrations. A horizontal red line divides the map into two regions. Upper region: The Martin–Hopkins equation is superior, with %Delta values being at least 5% less biased compared to the Friedewald equation. Lower region: The Friedewald equation is non-inferior, with less than a 5% difference in %Delta values. This heat map facilitates pattern recognition according to the hypothesis outlined in Table 3: the Martin–Hopkins equation tends to be highly inaccurate when LDL-C is low, triglycerides are low, and HDL-C is high. For detailed statistical analysis supporting these patterns, refer to Table 4.

To validate the perturbing factors shown in Table 3, we analyzed all individuals in which at least one of the three %Delta values was considered critical, defined as either below −20% or above 20% (see Section 2—Materials and methods. Initially, 40 cases were identified. After careful analysis, nine cases were excluded due to Lp(a) levels above 30 mg/dL to minimize interference, as Lp(a) is known to affect LDL-C estimations [9,10,11,12]. One case of critical LDL-C overestimation was also excluded: TC 115.7 mg/dL, dLDL-C 48.0 mg/dL, HDL-C 11.7 mg/dL, TG 246.8 mg/dL, %Delta Friedewald 13.9%, %Delta Martin–Hopkins 45.3%, %Delta Sampson 32.3%. Consequently, the remaining 30 cases were all instances of severe underestimation of the LDL-C concentration. Given that the primary focus of this study is the Martin–Hopkins equation as a personalized medicine tool, the 30 cases were ranked by their %Delta Martin–Hopkins values from the least to the most significant bias (%Delta). The data were then organized into a visually representative heatmap, as shown in Figure 3. Lastly, correlation analyses were performed for this subgroup of 30 individuals between %Delta values and blood lipid levels. These correlations were then compared with those performed on the entire cohort (see Table 4).

## 4. Discussion

### 4.1. Current LDL-C Measurement Methods

The reference method for the quantification of lipoproteins is beta-quantification, which requires ultracentrifugation. This method is expensive, time-consuming, and impractical for routine clinical practice. As an alternative, direct LDL-C measurement enzymatic and immunoassay kits are available. While the enzymatic method is well established, the accuracy of the immunoassays remains under scrutiny. Nevertheless, the enzymatic method has its own limitations. Many enzymatic colorimetric assays do not distinguish between cholesterol from LDL and Lp(a) particles. Thus, lipoprotein(a) is a frequently overlooked factor which may cause falsely elevated LDL-C levels in patients with high Lp(a), potentially resulting in unnecessary or inappropriate intensification of lipid-lowering therapy. To avoid additional costs, many clinical laboratories opt to calculate the LDL-C concentration using the Friedewald equation, which was first published in 1972 [1]. Since then, and especially in the last decade, numerous other equations have been proposed for the estimation of LDL-C based on standard lipid profile parameters. Some of these equations are derivatives of the Friedewald equation, while others employ different approaches, with some not even utilizing all three lipid parameters (TC, HDL-C, TG).

Given the superiority of dLDL-C assays, a legitimate question arises: why not measure LDL-C directly, especially in the context of precision medicine, rather than relying on calculations? However, the answer is not straightforward. Firstly, employing direct LDL-C (dLDL-C) assays for routine lipid profile testing incurs additional work and, most importantly, significant costs, which not all laboratories or healthcare systems can afford. Depending on the country, institution, or available funding, some laboratories may use dLDL-C for all samples, while others reserve it for cases where the calculated LDL-C is likely to be inaccurate. However, many laboratories have not implemented dLDL-C testing, primarily due to financial constraints, particularly in low-income and developing countries, although this is not uncommon in more developed settings as well. While we were unable to find reliable evidence from surveys, the Friedewald equation reportedly remains the most widely used method for estimating LDL-C, including in Western countries.

Secondly, as mentioned earlier, dLDL-C assays are not without their limitations. And thirdly, despite the known LDL-C underestimation by current equations, most of the clinical evidence informing current cardiovascular risk management guidelines is based on trials that used LDL-C values estimated by the Friedewald equation. Deviating from this method leaves clinicians in a gray area, where even though they may have more accurate LDL-C measurements, they lack established references for risk assessment. This presents a significant barrier to adopting newer methods, and it has been argued that Friedewald-estimated LDL-C should be preferred over dLDL-C for consistency with existing guidelines. Nevertheless, while the Friedewald equation has been an invaluable tool for decades, it should not hinder progress in refining cardiovascular risk assessments. We believe that the next decade will see an increase in clinical trials utilizing more precise LDL-C measurement methods, leading to a shift in guidelines and new thresholds based on these improved techniques.

### 4.2. What Is the Best LDL-C Estimation Equation?

A recent analysis of a large database comprising over five million patients compared the accuracy of 23 published equations [3]. The study found that the equations with the highest rates of correct LDL-C classification were Martin–Hopkins (89.6%), Sampson (86.3%), Chen (84.4%), Puavilai (84.1%), Delong (83.3%), and Friedewald (83.2%) [3]. The other equations performed worse than Friedewald, with classification accuracies as low as 35.1% [3]. Despite the existence of several proposed equations, some of which are evidently superior, the Friedewald equation remains predominant in clinical laboratories and is by far the most commonly used method for LDL-C estimation.

The Martin–Hopkins equation is a derivative of the Friedewald equation, where the fixed ratio TG/5 is replaced by a variable ratio, TG/F. The variable factor F is chosen from a table containing 180 cells based on the patient’s TG and non-HDL-C levels. This represents a significant advancement from traditional LDL-C estimation formulas that sought to determine the best fixed mathematical equation. A “one size fits all” approach may correctly estimate LDL-C levels for many patients, but it often fails in particular cases. Most notably, the traditional Friedewald equation is less accurate at low LDL-C levels, especially when TGs are elevated [1,2,4,5,6,8]. The Martin–Hopkins formula was specifically designed to address the inaccuracies of the Friedewald equation in these conditions [8]. This is particularly relevant in an era characterized by highly efficient lipid-lowering therapies and an increased prevalence of obesity, metabolic syndrome, and diabetes—all conditions associated with hypertriglyceridemia. Consequently, low LDL-C levels and elevated TG levels are becoming more commonly observed in clinical practice.

While the Martin–Hopkins equation is indeed more robust at low LDL-C levels, it still has limitations. For instance, similar to the Friedewald equation, the Martin–Hopkins equation should not be used when TG levels exceed 400 mg/dL. In contrast, the Sampson equation was designed to be robust even in cases where TG levels are greater than 400 mg/dL [5]. Subsequently, an adjusted Martin–Hopkins equation was published, which accounted for hypertriglyceridemia, thereby extending its applicability to a broader range of patients [4]. This extended Martin–Hopkins equation was shown to be more accurate than the Friedewald or Sampson equations [4]. With all these equations available, the question of what equation clinical laboratories should use remains. Laboratory information systems, which automatically apply the LDL-C estimation equation, can host more than one equation and apply each equation depending on the patient’s characteristics. However, for simplicity and clarity purposes, it would be ideal if only one equation was used by the laboratory. Several studies have shown that the Martin–Hopkins equation is superior to the other equations [3,4,8]. In the present study, performed on a mixed cohort of 210 individuals, we also showed that the Martin–Hopkins equation was generally superior to the Friedewald and Sampson equations. 

### 4.3. Particular Cases of Critical Estimation Inaccuracy

The importance of accurate LDL-C estimation lies not in the absolute LDL concentrations, but in the cardiovascular risk category assigned to the patient based on those concentrations. Underestimation of LDL-C can lead to undertreatment, which arguably poses a greater risk than overtreatment in the context of lipid-lowering therapies. As already mentioned, the Martin–Hopkins equation achieves the highest rate of correct LDL-C classification (89.6%), marking a significant improvement over the Sampson equation (86.3%) and the Friedewald equation (83.2%) [3]. Nevertheless, approximately 10% of individuals are still misclassified by the Martin–Hopkins equation. This misclassification may not always stem from a substantial inaccuracy in the equation itself but rather from the patient’s actual LDL-C concentration being very close to a clinical decision threshold. In such cases, even a minor bias in the equation could lead to misclassification (e.g., an actual LDL-C of 72 mg/dL and a Martin–Hopkins estimation of 69 mg/dL). However, among the 10% of misclassified patients, at least some are misclassified due to unacceptable inaccuracies in the equation.

In the present study, we aimed to identify specific cases where the three investigated equations were critically inaccurate, arbitrarily defined here as %Delta values below −20% or above 20%. Based on correlation analyses between %Delta values and dLDL-C, TG, and HDL-C (Figure 1 and Figure 2), we hypothesized which conditions would favor the underestimation or overestimation of LDL-C by each equation (Table 3). We then selected a cohort subgroup (n = 30) in which at least one of the three %Delta values was critically inaccurate, resulting in significant underestimation of LDL-C. Figure 3 suggests that when two or three such conditions are present simultaneously in the same patient, even the Martin–Hopkins equation becomes unacceptably inaccurate, with negative %Delta values as low as −45.4%. The influence of extreme concentrations of LDL-C, TG, and HDL-C on %Delta was demonstrated by their significantly improved correlations with %Delta when the cohort subgroup was analyzed separately (Table 4). Specifically, the worst underestimation of LDL-C occurs when low TG, high HDL-C, and low LDL-C occur concurrently (Table 3, Figure 3). Among these factors, TG appears to exert the greatest influence. Conversely, although less common, a reversal of these conditions (high TG, low HDL-C, but not high LDL-C—see the biphasic correlation in Figure 1) may lead to an unacceptable overestimation of LDL-C, illustrating the robust mathematical relationship between %Delta and these two lipid parameters. In this study, only one such case of critical LDL-C overestimation was identified: TC 115.7 mg/dL, dLDL-C 48.0 mg/dL, HDL-C 11.7 mg/dL, TG 246.8 mg/dL, %Delta Friedewald 13.9%, %Delta Martin–Hopkins 45.3%, and %Delta Sampson 32.3%. In this instance, although dLDL-C was low, the combination of very low HDL-C and elevated TG resulted in very high positive %Delta values. This underscores the significance of TG and HDL-C, rather than LDL-C, as the primary factors influencing the inaccuracy of the Martin–Hopkins equation. This finding aligns with the design of the Martin–Hopkins equation, which was intended to be more robust in the context of low LDL-C levels.

### 4.4. Personalized Approach to LDL-C Estimation

The traditional Friedewald equation operates on a “one size fits all” principle. While this equation has served the medical field well and has its merits, advancements should be made toward personalized LDL-C estimation in the era of precision medicine. The Martin–Hopkins equation is the most accurate equation, which is likely due to its design as a tool of precision medicine. Although it is more complex to implement compared to the simpler Friedewald or Sampson equations, the Martin–Hopkins equation warrants full consideration by clinicians and laboratory professionals alike.

Nevertheless, despite the significant advantages of implementing the Martin–Hopkins equation, both clinicians and laboratory professionals should be aware of its limitations. Firstly, this equation should not be used when TGs exceed 400 mg/dL. In such cases, the extended Martin–Hopkins or the Sampson equation may be employed, but even then, it is recommended to opt for a direct LDL-C measurement method [4,5]. Secondly, as demonstrated by the present study, the Martin–Hopkins equation can severely underestimate LDL-C in specific situations, such as when LDL-C and TG levels are low, and HDL-C levels are high. Thirdly, although less common, the Martin–Hopkins equation can severely overestimate LDL-C if TG levels are high and HDL-C levels are low, particularly if LDL-C is not low. Such scenarios, where factors influencing the equation’s bias align in the same direction, should be carefully acknowledged and managed by laboratory professionals.

**Figure 4:**
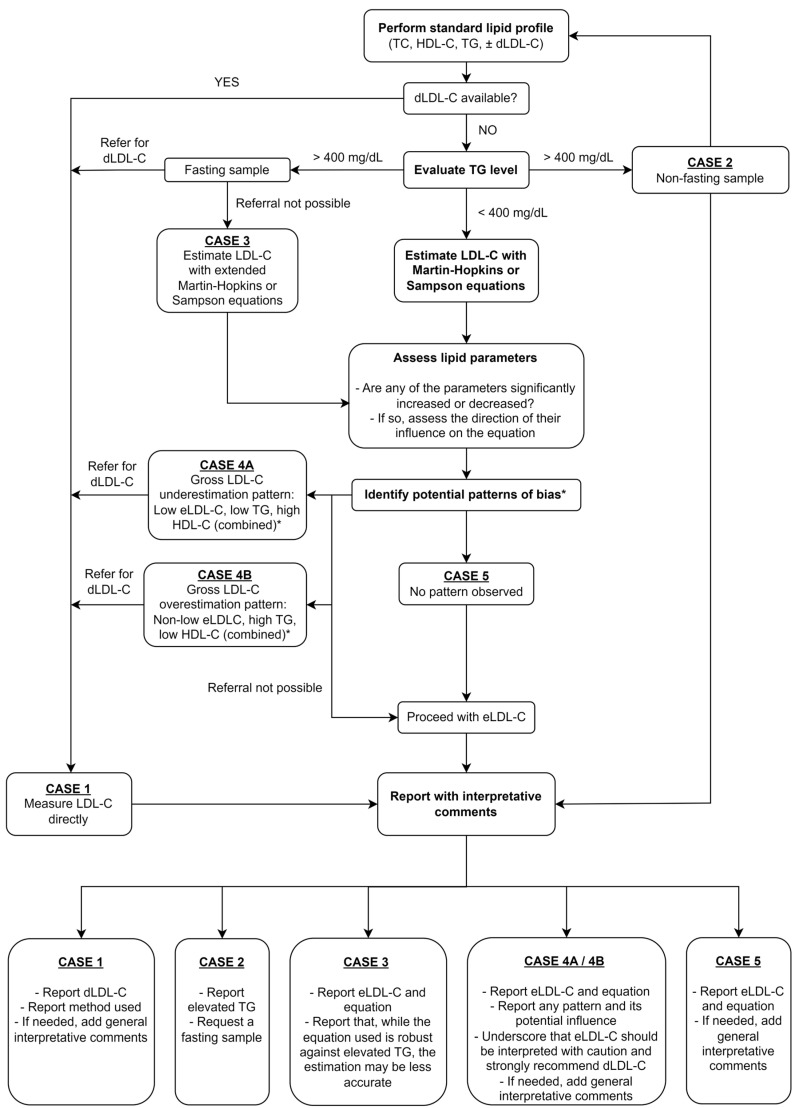
Proposed algorithm for personalized lipid management. * Note: Gross under/overestimation does not always require all three factors (significant skew in one or two factors can also lead to bias, especially if not counterbalanced by the other parameters). Case 3 can be combined with cases 4A, 4B, or 5. Abbreviations: (d/e)LDL-C—(directly measured/estimated) low-density lipoprotein cholesterol, HDL-C—high-density lipoprotein cholesterol, TC—total cholesterol, TGs—triglycerides.

Taking all these factors into account, we propose an algorithm for clinical laboratories to assess LDL-C concentrations in a personalized manner (Figure 4).

## 5. Conclusions

We live in an era of highly effective lipid-lowering therapies that achieve very low levels of LDL-C, coupled with an increased prevalence of hypertriglyceridemia due to obesity, metabolic syndrome, and diabetes. Therefore, transitioning from the traditional Friedewald equation to more accurate and robust methods such as the Martin–Hopkins equation has become a critical obligation rather than a matter of personal preference. The superior performance of the Martin–Hopkins equation has been consistently demonstrated, and our study further substantiates this claim. However, even the Martin–Hopkins equation is not without its limitations, particularly when multiple concomitant factors converge to skew its bias in the same direction. In this study, we identified patterns associated with significant underestimation of LDL-C by the Martin–Hopkins (and Sampson) equation and addressed these limitations. Based on our findings, we have proposed an algorithm for clinical laboratories to accurately assess LDL-C concentrations.

## Figures and Tables

**Table 1:** Measured and estimated LDL-C concentrations.

	dLDL-C	LDL-C Friedewald	LDL-C Martin–Hopkins	LDL-C Sampson
Mean	118.6	105.1	108.4	108.0
SD	39.0	37.9	37.0	37.6
Range	32.2–249.6	18.6–220.6	18.6–220.3	18.4–222.2
Delta: mean	Reference	−12.2	−8.8	−9.5
Delta: SD		9.2	9.8	9.2
Delta: range		−45.4 to 13.9	−45.4 to 45.3	−46.0 to 32.4

LDL-C mean, SD, and range are expressed in mg/dL. Delta mean, SD, and range are expressed as percentages (%). Abbreviations: dLDL-C—directly measured low-density lipoprotein cholesterol, SD—standard deviation.

**Table 2:** Variation in %Delta values depending on statin treatment or LDL-C concentration subdomain.

	No Treatment (n = 120)				Statin Treatment (n = 90)			
	dLDL-C	Friedewald	Martin–Hopkins	Sampson	dLDL-C	Friedewald	Martin–Hopkins	Sampson
Mean	126.3	115.6	117.5	118.0	108.4	91.2	96.3	94.7
SD	34.0	32.9	32.8	33.0	42.8	39.7	38.9	39.4
Range	34.1–218.9	18.6–218.8	18.6–220.3	18.4–219.9	32.2–249.6	22.2–220.6	21.6–219.3	21.0–222.2
Delta: mean	Reference	−8.8	−7.1	−6.8	Reference	−16.8	−11.1	−13.1
Delta: SD		8.0	8.7	8.2		8.7	10.8	9.2
Delta: range		−45.4 to 6.6	−45.4 to 20.9	−46.0 to 12.4		−34.8 to 13.9	−32.8 to 45.3	−34.7 to 32.4
	**dLDL-C ≤ 130 mg/dL (n = 135)**				**dLDL-C > 130 mg/dL (n = 75)**			
	**dLDL-C**	**Friedewald**	**Martin–Hopkins**	**Sampson**	**dLDL-C**	**Friedewald**	**Martin–Hopkins**	**Sampson**
Mean	95.4	83.1	87.0	86.1	160.4	144.9	146.9	147.5
SD	21.8	23.5	23.1	23.5	26.2	23.6	23.9	23.4
Range	32.2–129.0	18.6–130.7	18.6–144.3	18.4–135.0	130.7–249.6	111.7–220.6	111.4–220.3	112.6–222.2
Delta: mean	Reference	−13.8	−9.2	−10.4	Reference	−9.4	−8.2	−7.8
Delta: SD		10.2	11.1	10.3		6.2	6.9	6.3
Delta: range		−45.4 to 13.9	−45.4 to 45.3	−46.0 to 32.4		−21.4 to 4.4	−19.8 to 15.2	−19.1 to 7.9

LDL-C mean, SD, and range are expressed in mg/dL. Delta mean, SD, and range are expressed as percentages (%). Abbreviations: dLDL-C—directly measured low-density lipoprotein cholesterol, SD—standard deviation.

**Table 3:** Factors associated with high or low LDL-C estimation bias.

		Friedewald	Martin–Hopkins	Sampson
Extreme LDL-C underestimation	LDL-C	↓	↓	↓
	TG	NSA **	↓	↓
	HDL-C	NSA	↑	NSA
Minimal LDL-C underestimation *	LDL-C	↑	NSA	NSA
	TG	NSA **	↑	↑
	HDL-C	NSA	↓	NSA

* Less commonly, cumulated factors can result not in minimal LDL-C underestimation, but in overestimation (sometimes significant). ** In the entire cohort, the correlation between %Delta Friedewald and TG levels was not significant (see Figure 2). However, TGs are associated with significant underestimation of LDL-C when the LDL-C levels are low. Specifically, in the present study, the Friedewald equation showed a significantly lower degree of inaccuracy (median Delta −10.3%) when dLDL-C levels were >100 mg/dL and TG levels were ≤150 mg/dL. In contrast, when dLDL-C levels were ≤100 mg/dL and TG levels were >150 mg/dL, the degree of underestimation increased substantially (median Delta −20.4%, *p* = 0.0014). Abbreviations: HDL-C—high-density lipoprotein cholesterol, LDL-C—low-density lipoprotein cholesterol, NSA—not significantly associated, TGs—triglycerides.

**Table 4:** Particular scenarios: comparison of correlations between %Delta values and blood lipid concentrations.

	%Delta—Subgroup of Critically Underestimated LDL-C (n = 30)			%Delta—Entire Cohort (n = 210)		
	Friedewald	Martin–Hopkins	Sampson	Friedewald	Martin–Hopkins	Sampson
TG	r = −0.086 *p* = 0.64	r = 0.768 *p* < 0.0001	r = 0.483 *p* = 0.0068	r = 0.006 *p* = 0.92	r = 0.446 *p* < 0.0001	r = 0.202 *p* = 0.0032
HDL-C	r = −0.006 *p* = 0.97	r = −0.421 *p* = 0.020	r = −0.230 *p* = 0.22	r = −0.029 *p* = 0.67	r = −0.281 *p* < 0.0001	r = −0.128 *p* = 0.063
dLDL-C	r = 0.661 *p* = 0.0001	r = 0.406 *p* = 0.026	r = 0.613 *p* = 0.0003	r = 0.293 *p* < 0.0001	r = 0.072 *p* = 0.29	r = 0.189 *p* = 0.0059

For individuals selected in the subgroup (n = 30, see Figure 3), at least one of the three Delta values was considered critical, defined as either below −20% or above 20%. The significantly stronger correlations in the subgroup compared to the entire cohort confirm the dramatic cumulative effect of the Delta-influencing factors (LDL-C, TGs, and HDL-C) on the accuracy of the Martin–Hopkins and Sampson equations, as outlined in Table 3. Abbreviations: dLDL-C—directly measured low-density lipoprotein cholesterol, HDL-C—high-density lipoprotein cholesterol, TGs—triglycerides.

## Data Availability

The raw data supporting this study’s findings are openly available in FigShare at https://doi.org/10.6084/m9.figshare.26391265.
